# Analysing the interactions and complexities of the operations in the production area of an FPSO platform using the functional resonance analysis method (FRAM)

**DOI:** 10.1007/s12517-022-09801-0

**Published:** 2022-03-19

**Authors:** Josué E. M. França, Erik Hollnagel, Gesa Praetorius

**Affiliations:** 1 Linnaeus University, Växjö, Sweden; 2 University of Jönköping, Jönköping, Sweden; 3 University of South‐Eastern Norway, Notodden, Norway

**Keywords:** FPSO, Production, FRAM, Safety, Human factors, Non-technical skills

## Abstract

The technological evolution of several productive sectors of society has demanded the same level of evolution for the oil and gas industry, both for energy production and their own systems’ functioning. The production of crude oil and natural gas in offshore units is one of the answers to this demand. However, these offshore units have critical onboard activities and risks, notably FPSO units; it is necessary to have adequate recognition of the elements that can support these activities and manage these risks, enabling productive and safe operations. In this sense, this article aims to increase the understanding of the complex interactions and inherent safety issues that arise in the operations of FPSOs, observing and analysing the work done onboard such platforms. The FRAM methodology has been chosen because it allows for the recognition and analysis of the complex interactions involving workers, equipment, system and offshore environment, focusing on the oil treatment area of the process plant. The results demonstrated some interesting findings regarding onboard safety and the relationship between human competences, work demands and process safety.

## Introduction

Based on the 69^th^ edition of the *Statistical Review of World Energy* published in 2020 by British Petroleum (BP), world oil consumption has grown by 0.9 million barrels per day (b/d), or 0.9% slightly lower than the 10-year average of 1.3% p.a. In this scenario, Brazil produces 2.877 thousand barrels per day, and more than 80% of this comes from offshore production. Brazil is currently the 10th largest oil producer in the world and the largest in Latin America (Petersohn 2019). So far, the country’s development and consumption needs are being met by its production and reservoirs, which increasingly demands the construction and deployment of new offshore production platforms, further away from the coast, in ultra-deep waters, and there is a pressing need to store the oil produced. This has created a perfect scenario for the FPSO units.

**Fig. 1:**
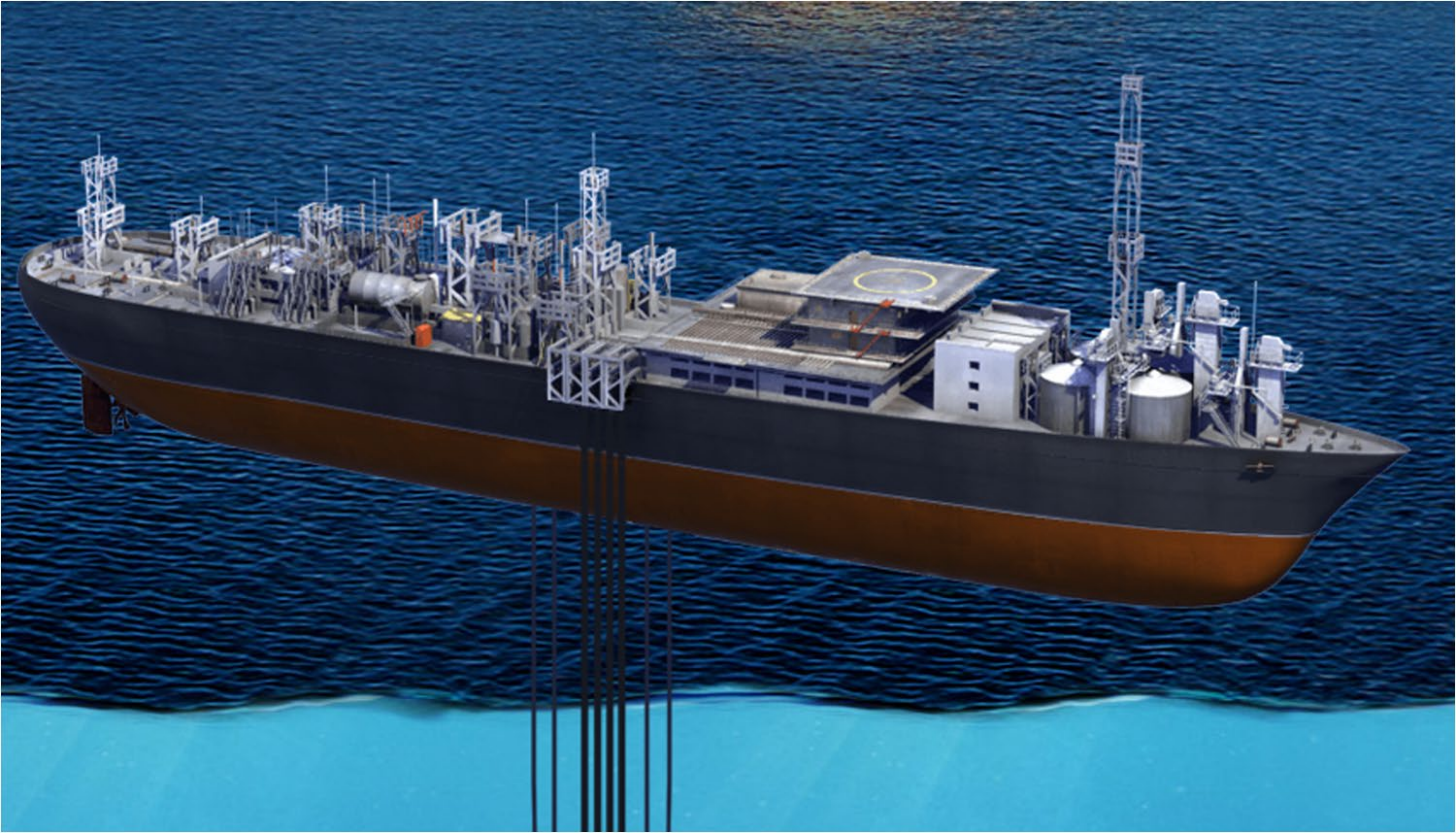
Illustration of an FPSO (floating, production, storage and offloading) platform. Source: Petrobras (2020)

A floating, production, storage and offloading (FPSO) unit is an offshore platform unit that combines an oil and gas (O&G) production platform and an oil tanker vessel. This type of hybrid platform can be designed by having their hull, tanks and production systems built from scratch as an FPSO. In other cases, they are single-hull oil tankers that are converted: these ships return to the shipyards and are decontaminated and adapted for an O&G production plant to be installed from the stern to the bow, taking advantage of some vessel systems, such as stability, living quarters, utilities and, especially, oil tanks. FPSO units are widely used in offshore productions areas far from shore, in deep and ultra-deep waters, where the construction of pipeline infrastructure has technical and economic limitations. This platform is responsible for the primary processing of oil and other complementary hydrocarbons treatments for expedition and offloading (Allahyarzadeh-Bidgoli et al. 2018). Considering the relevance of the FPSO units for the Brazilian O&G business, especially in the pre-salt province, the development of safe and efficient operations is essential from the very beginning of the life cycle of an FPSO. Figure 1 presents an illustration of this platform.

This hybrid unit is composed of elements of a ship – stability, anchoring and hull systems; as well as elements of an offshore platform – crude oil production and processing operation systems. It concentrates a series of risks related to the vessel with oil and gas production, integrating these two distinct engineering systems. Offshore workplaces at FPSO units and oil rigs (Baksh et al. 2016), as well as at wind farms (Buchana and McSharry 2019), have systems and equipment running at the edge of technology, pushing process parameters such as temperature, pressure and torque to their critical limits. Systems that work with such parameters under extreme conditions generate highly complex variables in their functioning, having interactions at multiple scales (de Bruijn et al. 2017), which increases interactions, complexities and risks. From this, drilling incidents such as sticking, nozzle clogging and mud loss (C. Wang et al. 2020) can emerge, demanding complex operations and advanced equipment for well control (Sule et al. 2018). Natural gas blowout, from drilling or production wells, when out of control, can cause great harm to the platform operators, facilities and environment (Wei and Geng 2021). FPSO-type production platforms, which have a substantial oil inventory in their storage tanks, are a potential risk of major accidents and environmental disasters (Vinnem 2018). Accidents in offshore work environments, due to their complexity and location, in addition to immediate consequences, generate medium and long-term impacts on marine biodiversity (Yang et al. 2020). Also, as in many other high‐risk domains, offshore safety regulatory institutions demand solutions and apply consequences for incidents and accidents in these workplaces (G. Praetorius et al. 2020). Therefore, it is necessary to develop a systemic and integrated approach to all these elements in order to understand and analyse the interactions of this complex sociotechnical workplace. Based on that, the hypothesis that traditional risk analysis methodologies do not have a structure capable of dealing with the complexities that emerge was developed, enabling the search for solutions that deal with all of this in an integrated manner. To this end, this research applies the functional resonance analysis method (FRAM), aiming for a comprehensive understanding of how the work is done in such places.

## Characteristics and competences of the offshore work

The interaction between the workers and the sea in the West is ancestral, with historical reports of small sailing boats that managed to reach kilometres of distance between continents in 1500 BCE (Guy 2014). Notably, during the First and Second World Wars, the evolution of technology was accelerated by needs due to the exchange of engine power from coal to liquid fuels – gasoil, bunker and others. The most striking of these developments was promoted initially by the British Empire but followed by the other countries involved in the conflict (Yergin 2008). Right after the Second World War, the world’s oceans were restrained by the fleets of the traditional maritime nations, predominantly the USA, France, Holland, the British Empire and the Scandinavian countries (Grech et al. 2008). Despite the notable evolution of the technology in the engineering systems that are placed in the sea – ships, platforms, wind generators – there is an element that remains the same in terms of its evolution but is the driving force of all that: The workers who project, build, operate and maintain all these systems.

In this context, seeking a balanced understanding of how offshore workplaces function, analysing their limitations and capabilities through their own technical evolution requires developing a comprehension not focused on the workers’ flaws. Instead, it is necessary to understand the limitations, capabilities and natural preparedness that keep them productive in their work routines and prepared for emergencies and contingencies. A human factors approach (França et al. 2020), where a systemic and integrated analysis is conducted to understand the elements that may have an influence on the workers’ performance, is one way to have this equalised understanding of complex sociotechnical offshore systems. In this context, some specific competences and skills play an important role, in being responsible for various interactions in the workplace. These interactions, in addition to marking the difference between the work as imagined (WAI) and the work as done (WAD), enable the entire system to be prepared for expected and unexpected responses.

Specifically for the O&G area, these workers’ competences have been classified as non-technical skills, which are defined as the cognitive and social skills that complement technical skills, contributing to the safe and efficient performance of the work (Flin et al. 2016). Five of them are often highlighted due to their importance in the context of work: communication, leadership, teamwork, decision-making and situation awareness. The recognition of non-technical skills in safety and performance has already been applied in industries characterised by complex workplaces, such as civil aviation, nuclear, railroads and healthcare (Thomas 2018). Contextualising such recognition in offshore work – platforms, oil tankers, etc. – the O&G industry noticed that competences not only are present in such workplaces but are also responsible for onboard safety performance (IOGP 2020). Indeed, sharp-end knowledge is a structuring element of the system’s resilience, relying on the operator’s skills for continuous learning (Patriarca et al. 2021). Onboard an FPSO unit, especially in the production plant, there is an intense interaction between workers, equipment and systems; the recognition and analysis of non-technical skills is fundamental for safe and productive activities.

## Materials and methods

For the development of this research, four distinct stages were adopted, considering the description of the observed work, the methodology and its application, as well as the analysis and discussions. These four steps are as follows.

• Step 1 – Description of the FPSO operations.

• Step 2 – Description of the FRAM methodology.

• Step 3 – Development of an FRAM model of the FPSO operations under study.

• Step 4 – Analysis and discussions on the variability of the FRAM model.

### Analysing FPSO operations of oil treatment

A typical FPSO production system is composed of a complex underwater structure of producing wells controlled by wet Christmas tree (WCT) and manifolds (sets of valves and connections) connected to a topside process plant, placed on the main deck of the FPSO, which is responsible for the processing, storage and offloading of all the oil and natural gas produced (Abramowski 2006). The transfer of oil from the FPSO to floating, storage and offloading (FSO) relief vessels is also part of the production process – This unique operation is the offloading. The natural gas is transferred from the FPSO to special LNG ships or pipelines. The topside process plant is composed of two trains of production, each containing processing equipment such as heat exchangers, production separators, electrostatic dehydrators, oil handlers and atmospheric separators (Q. Wang et al. 2010).

**Fig. 2:**
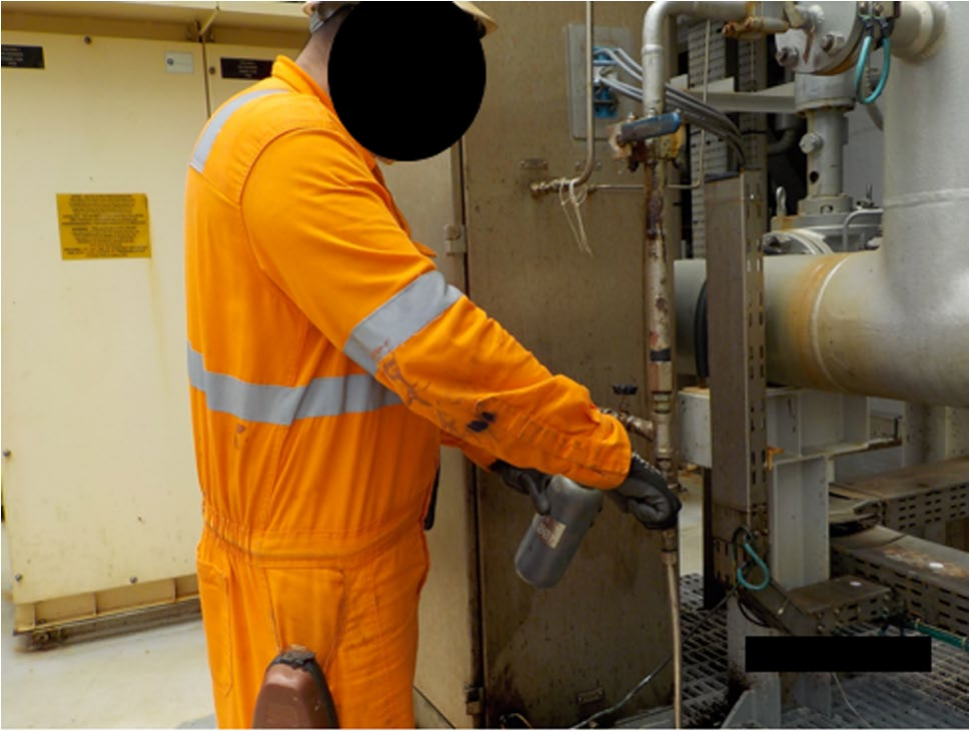
Operator performing an oil sampling operation in the process plant. Source: Authors (2020)

The production separator has the function of removing the sand contained in the oil through filtration. The separated oil goes to the electrostatic dehydrators, where there is a maximum separation of salts and water from the oil, also called BSW. The dehydrated oil is cooled in the heat exchangers, which exchange heat with seawater. The oil is then stabilised in the atmospheric separators, where there is an oil/associated gas separation, and the oil is stored in the cargo tanks till further transfer in the offloading operations (França 2014). The separated natural gas goes to the gas processing plant; once processed, it goes to power generation and FPSO utilities or is transferred for ships and pipelines or relieved on the flare. Figure 2 shows an operator performing an oil sampling operation in the atmospheric separators of the oil treatment area of the process plant. It is a typical and routine onboard activity of FPSO production operators.

The onboard operators perform a series of tasks to ensure the continuous operation of the production system by monitoring, controlling, acting and interacting with different variables, equipment and people in different processes and layers of the entire platform – a complex sociotechnical system. The most critical process variables of an FPSO are present in the production area – the higher temperatures, pressures, flows and inventories. Moreover, petroleum itself is already a substance that damages human health and is extremely flammable, presenting an average flashpoint of the order of − 7 ℃. The production pressure, which is the flow of petroleum from the well to the topside process plant, in deepwater reservoirs, can range from 10 up to 100 kgf/cm^2^ (Ahmed Ali et al. 2019), depending directly on the reservoir pressure.

**Fig. 3:**
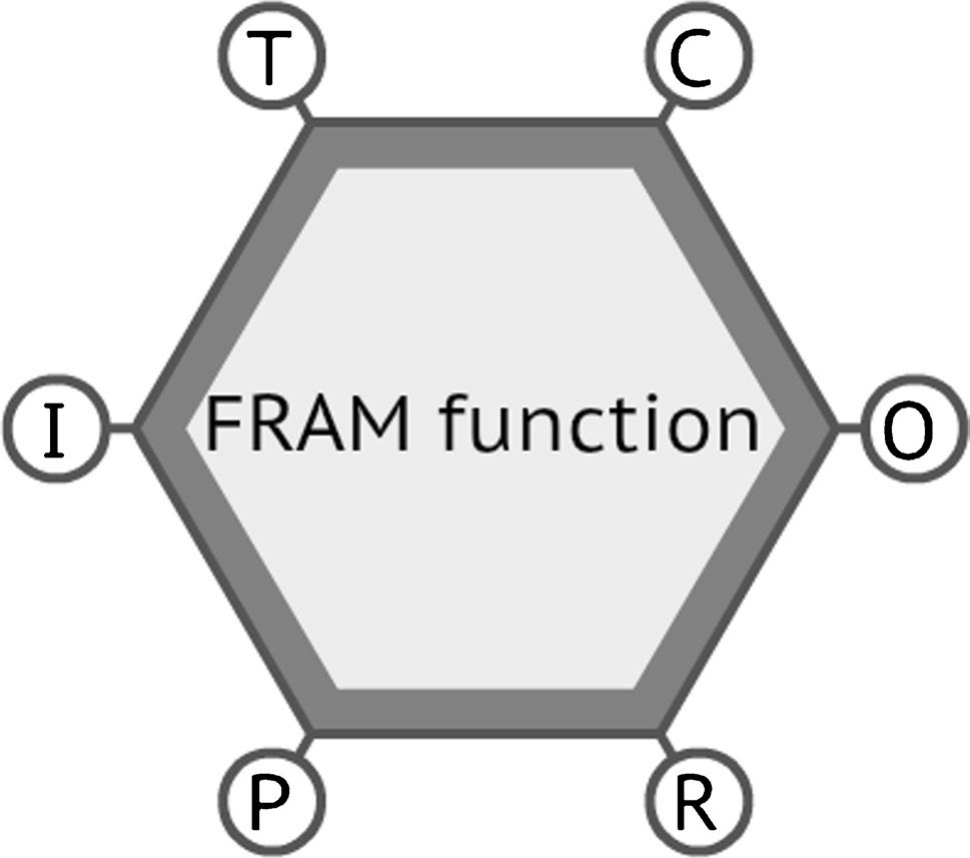
Representation of an FRAM function with its six aspects. Source: Authors (2020)

Besides the external operator’s activities in the process plant (see Fig. 3), there are also internal activities in the central control room (CCR), a workplace where a high interaction between different areas occurs, once the operators of the production, facilities and stability areas are in the same place. Additionally, in this place, there are professionals of maintenance and instrumentation, as well as the supervisors and coordinators of all FPSO areas. These professionals and managers are not the fixed crew of the CCR; however, they need to be there, and they interact directly with operators in two different work shifts. A typical workday on an offshore platform involves a 12-h shift with two small breaks, plus a lunch. Operators work in this 12-h shift, with a rest period of an equal 12 h, covering the 24-h non-stop operations of the platform. In some cases, it is possible to work a mix of day shifts and night shifts, depending on the scheduling, sea conditions or state of operations. Observing the onboard activities of the production operator, in their different shifts, reveals that they perform several tasks. Of these tasks, the most frequently executed in their daily routine are as follows:

• shift change briefing between operators (leaving for rest and others coming to work);
• utilisation of communication devices to request information, services, instructions etc.;
• utilisation of the radio to communicate with operators who are in the external area;
• interaction, monitoring and interpretation of several control screens of the production supervision system;
• from the external area, communication by radio with other operators inside of the central control room;
• emission, authorization and creation of work permits (WP) for maintenance and other activities in the operational external area;
• operation (starting, stopping and monitoring) of pumps in the external area;
• daily checklist inspection of the entire external area of the process plant;
• sampling of oil, water and gas from the process plant equipment;
• local verification of level measurement, through observation of equipment level gauge (LG);
• verification of pressure measurement through observation of equipment pressure indicators (PI);
• interaction with maintenance, instrumentation and inspection professionals carrying out activities in the external area;
• interaction with service providers, carrying out activities in the external area
• water drainage, gas and oil condensate from instruments, equipment, pipelines and accessories;
• manual valve manoeuvres to perform alignments for routine tasks, maintenance, contingency or emergency; and
• operational assistance for emergencies that may occur, performing operator or fire brigade tasks.

These onboard activities observed by the researchers were validated by the operators themselves, who confirmed that these activities are the most routinely performed by production operators who work in the oil treatment area. Also, these activities are the basis for the FRAM model presented in this research. This study is limited to oil treatment activities in the processing plant of the production operation area. Other activities in the production area, such as offloading, as well as stability and facilities operations, will not be the object of this research and are potential areas for future studies.

### The FRAM methodology

The workplaces of modern industries that work in the limits of engineering demand a wider comprehension of the risk and safety management boundaries, which arise from the evolving nature of technology and the competitive, fast-moving modern working conditions (Patriarca et al. 2021). Onboard risks in the process plant of offshore facilities involve complex system elements interactions, including workers, equipment, organisational culture and information flow, as well as nonlinear coupling relationships among them. In this context, offshore work activities have been analysed by traditional risk analysis using machine learning algorithms (Zhang et al. 2018), dynamic Bayesian network (Sule et al. 2018) and custom computer simulations (S. Wang et al. 2021) that can handle complicated technological system, but still lack an appropriate approach to complex sociotechnical systems. Traditional risk assessment methodologies focus on a linear cause and effect relationship between the system elements, ignoring how the interactions and outcomes happen in real work scenarios (W. Li et al. 2019). Offshore activities, such as drilling and production, involve critical operations at sea with process equipment working at the limits of their parameters, demanding increasingly complex interactions in the control of the entire system (Mardanirad et al. 2021). Looking to understand the interactions and outcomes in real work scenarios, the methodology applied in this research is the functional resonance analysis method (FRAM), which enables comprehensive modelling of how work activities are done by considering human interactions and system complexity. The qualitative analysis delivered by this methodology does not involve heavy math calculations or complex concepts, although can concisely model the nonlinearity of a complex sociotechnical system in any moment or instantiation of its functioning. This nonlinearity is an essential characteristic of complex system, consisting of numerous interacting components that affect the system itself and are affected by one another (Tian and Caponecchia 2020). This set of characteristics allows for an adequate analysis of non-technical skills, human factors and complexities of FPSO production operations.

The FRAM methodology is structured by four principles (Hollnagel 2012). First, there is an equivalence of failures and successes, where failures, accidents and regular everyday work have the same origin. Second is the principle of approximate adjustments, where individuals or workgroups dynamically adjust their everyday performance to be able to respond to the complex demands of the system. Third is the principle of emergence, where events appear to be an emergent – dynamic and nonlinear combination of time, space and singular characteristics – rather than resulting from a specific combination of fixed conditions in a linear flowline. Fourth, there is functional resonance, which is the reverberance of the system’s complexity on itself, like a detectable signal emerging from the unintended interaction of the everyday variability of multiple signals. It is noteworthy that the resonance is not fully stochastic since the variability of the signals is not completely random, as they meet certain regularity parameters (Hollnagel 2012).

To develop FRAM modelling, it is necessary to follow particular steps and observe some boundaries. The graphic representation of the model’s functions is quite different from other methodologies, being a hexagon, where each corner has a different meaning and, thus, a purpose. Each corner determines one of the six aspects of an FRAM function: time, control, output, resource, precondition and input (Hollnagel et al. 2014). Also, each function can be classified differently from each other – as human, technological or organisational, depending on its nature – considering the sociotechnical system under analysis. An FRAM function describes how a task is really done, considering the everyday constraints of the workplace and the human interactions within (Hollnagel et al. 2014). Therefore, it allows an adequate recognition of the WAD versus the WAI, considering the real and dynamic elements of a working system. Figure 3 represents an FRAM function, with its six aspects and the unique hexagon format.

The FRAM methodology considers how both negative (unexpected) and positive (expected) events are the natural results of the variable combinations of the complex sociotechnical systems that comprise the workplace (Saldanha et al. 2020). This makes the analysis provided by the FRAM a systematic understanding of how things work – whether it is regarding accidents resulting from improvisation in workplaces, aircraft maintenance activities, construction site analysis or shoe manufacturing (Tian and Caponecchia 2020). As such, the model developed by this methodology can be considered as a map to understanding real work practices and their variabilities, describing the WAD and its contribution to safe everyday operations (De Vries 2017). The software FRAM Model Visualizer (FMV®) (Hill 2018) was utilised to build the FRAM model for this research, setting and connecting the function’s aspects into its couplings based on the evidence of the onboard WAD observed and analysed. Further validation by the operators and other necessary alterations were also conducted using this software.

### Building the FRAM model

The initial step in building an FRAM model is observing the real work done by the operators. Based on previous research on FPSO platforms operations (Wang et al. 2010; França 2014; Luquetti dos Santos et al. 2020), a series of five onboard observations were performed in five different months, accompanying the work of five different crews. Concomitant with these onboard observations, unstructured interviews were conducted with the operators who voluntarily participated in the research. During the observations, questions were not allowed so as not to hinder the work of the operators, as required by the platform managers. All the unstructured interviews were conducted after the operator’s 12-h shift, when they were leaving for rest. From these observations and interviews, a preliminary model was built.

The validation of the FRAM model by offshore experts, FRAM specialists and the operators themselves who participated in the initial stages of the research was conducted partly in person and partly online, as this step coincided with a few months of the restrictions imposed by the COVID-19 pandemic. Some validation meetings took place at the platform´s disembarking airport in the Jacarepaguá region of Brazil; the others were conducted using digital resources, such as e-mail, Facetime, Zoom and Microsoft Teams. The validation stage of the FRAM model is an important step because it must reflect the real work done by the operators, so they play an important role – along with offshore experts – in the verification of the time and precision variability of the main and most critical function outputs. Also, the validation done by FRAM specialists allows for adequate use of the method, engaging theory and appropriate practices for balanced research.

## Results and discussion

**Fig. 4:**
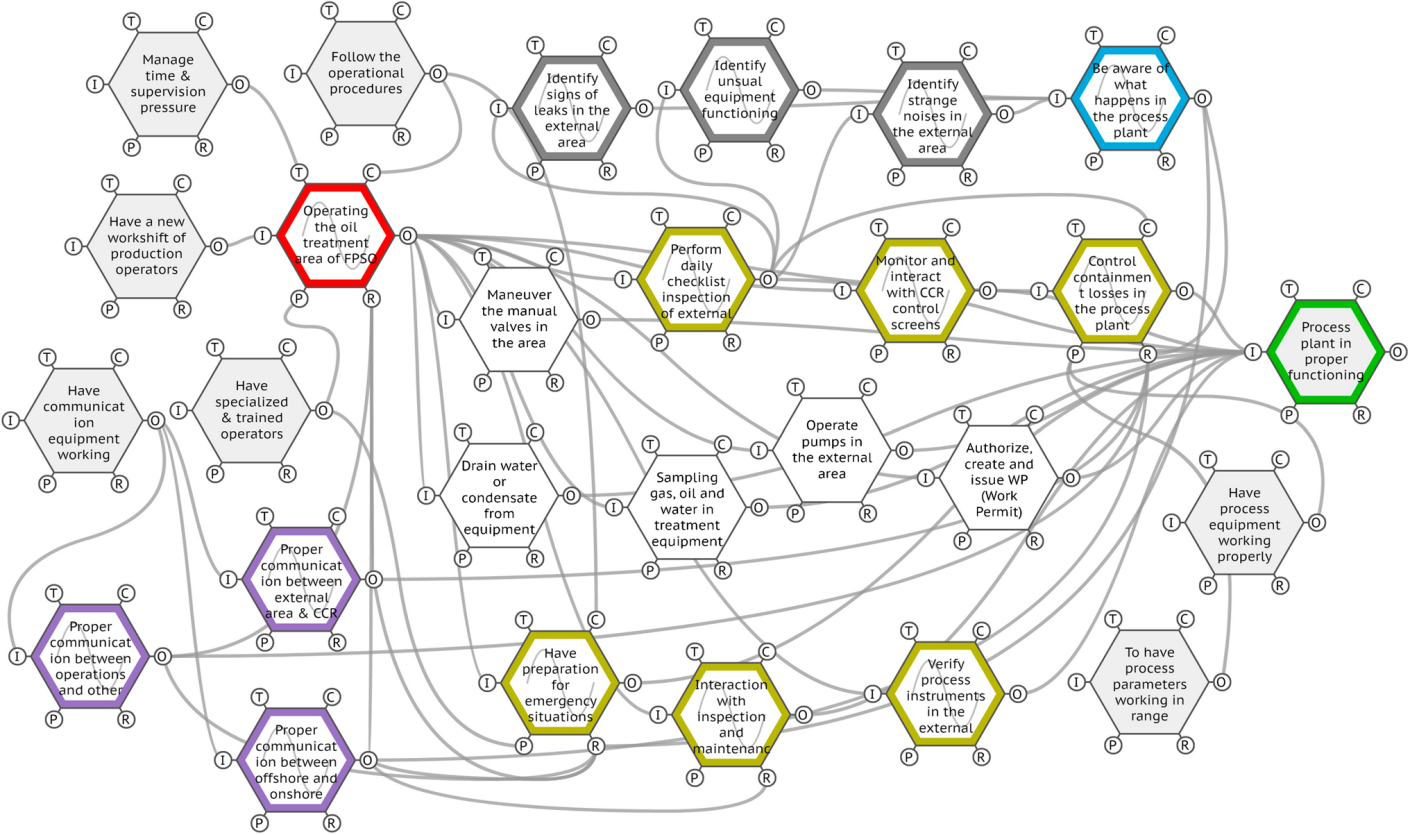
FRAM model of the FPSO oil treatment operations. Source: Authors (2020)

The FRAM model of the FPSO operations of oil treatment was built with 27 functions (see Fig. 4). From these 27 functions, seven were classified as background functions and 20 as foreground functions. Despite these classifications, some of the background functions are very strategic, as they are essential resources for the others. The foreground functions highlighted in colour are a key function in the analysis of the interactions and complexities of operations in the production area; they will be discussed thoroughly later on.

In this model, 14 of the 20 foreground functions presented output variabilities in terms of time and precision (see Table 1). Of these, five functions presented variabilities simultaneously in the categories of time and precision, which are highlighted in bold.

**Table 1:** FRAM model output variabilities. Source: Authors (2020)

Function	Variability	
	*Time*	*Precision*
**Operating the oil treatment area of FPSO**	**Too late**	**Acceptable**
Have preparation for emergency situations in operations	On time	Acceptable
**Verify process instruments in the external area**	**Too late**	**Imprecise**
**Interaction with inspection and maintenance teams**	**Too late**	**Acceptable**
**Monitor and interact with CCR control screens**	**Too late**	**Imprecise**
Control containment losses in the process plant	On time	Acceptable
**Perform daily checklist inspection of external area**	**Too late**	**Acceptable**
Identify signs of leaks in the external area	On time	Acceptable
Identify strange noises in the external area	On time	Acceptable
Identify unusual equipment functioning	On time	Acceptable
Be aware of what happens in the process plant	On time	Acceptable
Proper communication between operations and other areas	On time	Acceptable
Proper communication between external area and CCR	On time	Acceptable
Proper communication between offshore and onshore crew	On time	Acceptable

### Analysing interactions, complexities and competencies

Some outputs of the functions presented variabilities only in one of the categories of precision or time, which shows that there are some adjustments happening, but this could still be considered as being relatively under the boundaries of the designed job. However, some other outputs had variabilities in both categories, establishing a full variability in this output. It is important to note that this full variability in time and precision is not exactly a problem; it is also a reflection of adjustments and the natural human ability to respond to the system´s demands. In this case, the operators use their technical and non-technical skills, integrated with their individual characteristics, to assess, understand and respond to all of the FPSO’s process demands, regardless of whether they came from organisational requirements, equipment issues, human interactions or operational environment. The functions that presented variabilities simultaneously in time and precision will be closely examined to understand their role in the sociotechnical system.

#### The function ‘operating the oil treatment area of FPSO’

**Fig. 5:**
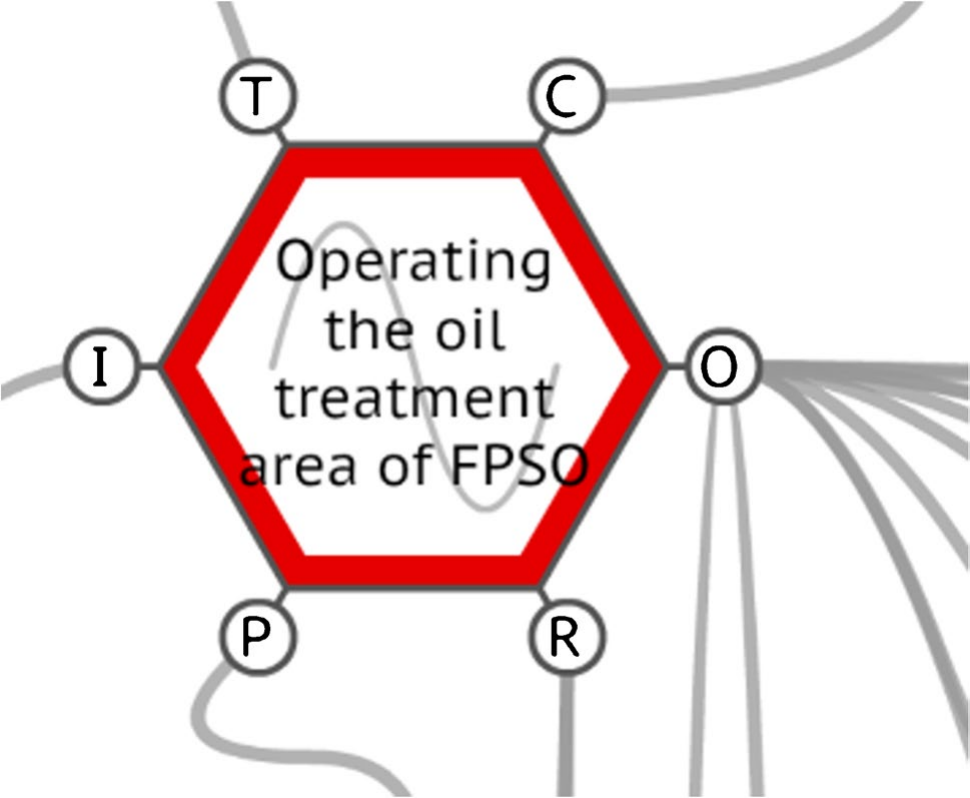
The FRAM function ‘operating the oil treatment area of FPSO’. Source: Authors (2020)

The function ‘operating the oil treatment area of FPSO’ presented the variabilities ‘too late’ for time and ‘acceptable’ for precision. It is the core function of the model, having eleven different outputs and connections in all aspects of the function, which can be seen in Fig. 5.

Also, this function is the representation of the operator’s work in real conditions, which is influenced by all human factors present in this job. Consequently, the variability in and from this particular function is under several internal and external influences; the operator is responsible for receiving all these inputs, managing them and responding to the demands of the sociotechnical system. This not only corroborates the difference between the WAD and the WAI, but it also allows for the comprehension of how the various outputs of this function are the actions that characterise the operator’s everyday work, preparing them for expected and unexpected situations. In fact, this ability to sustain the required functioning and achieve system goals under a variety of operational conditions can be understood as the resilience of the system (Praetorius et al. 2015). Increasing resilience, in this context, is expected to lead to a desirable outcome, bringing the system to a stable state – normative or not – preventing LOPC and other losses (de Bruijn et al. 2017).

The numerous tasks performed by operators both inside the CCR and in the process plant external area denoted by this function also highlight the consequences of this intense and multitasking work. Particularly in industries with sociotechnical complexity, such as civil aviation and the O&G offshore industry, elements that can cause human fatigue – like sleep deprivation, circadian rhythm abnormalities, health-related tiredness and task-induced influences – may have adverse effects on human performance (Bendak and Rashid 2020). These adverse effects may cause significant degradation in the workers’ interaction within the system, leading to unwanted onboard outcomes. In addition, in a sociotechnical system, such as the oil treatment area of an FPSO, the preparedness and knowledge on recovery counteractions reduce under degradation, but increase when there are certain levels of preparation and resources (de Bruijn et al. 2017).

This preparation for recovering from future critical events is something that can be attributed to resilience; it requires technical (i.e., equipment) and human resources (i.e., skills) to manage, adapt and maintain stability (Duchek 2020). In this sense, the onboard observations revealed that variabilities frequently occurred, with multiple tasks needing to be executed by operators under a limited amount of time or at the same time. In this function, the output variabilities are not something unwanted; on the contrary, it is something that allows the work to be carried out in an appropriate, productive and safe way, despite the constraints imposed by the operation of the system. This ability to deal with unexpected events in a complex system, responding productively to significant changes, can be directly associated with the organisational resilience of a company, something that nurtures not only safety but also business continuity (Duchek 2020). Other functions of this FRAM model that will be studied later on are intrinsically connected to this one; they recognise the cognitive and social competences necessary for an operator’s preparedness to promote the system’s resilience.

#### The function ‘verify process instruments in the external area’

**Fig. 6:**
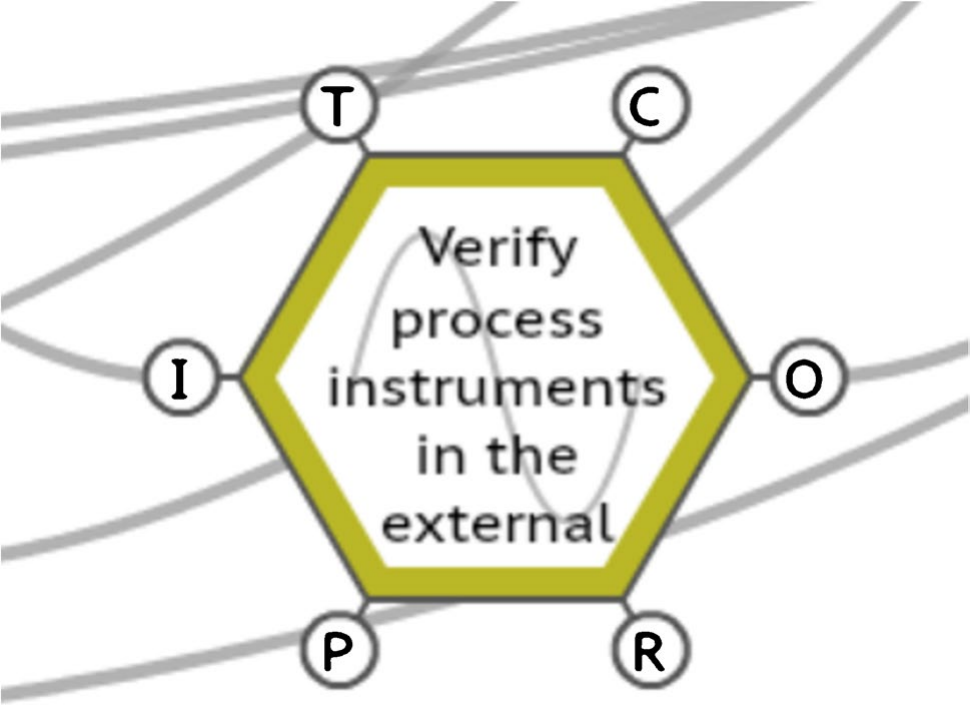
The FRAM function ‘verify process instruments in the external area’. Source: Authors (2020)

The function ‘verify process instruments in the external area’ presented the variabilities ‘too late’ for time and ‘imprecise’ for precision. Especially regarding imprecise variability, this function sets a warning regarding this activity. The variability induced by this function resonates through the system, contributing to the variability of other functions, as demonstrated by the model. This function is presented in Fig. 6.

The variabilities presented by this function in terms of precision being imprecise, as observed onboard and in the operator’s interviews, arise because of several instruments being damaged, due to the saltiness of the offshore environment, maintenance failures, impact with moving loads, natural wear or the improper actions of third parties. For instance, regarding improper actions, one of the operators reported that a scaffolding assembler screwed a tubular section of the scaffolding into a level metre, completely damaging it. In addition, imprecise output also results from erroneous readings presented by the instruments on the CCR control screens. It is often necessary to use the radio to ask the operator in the external area to read the instrument locally. However, in many cases, this reading is also impaired by ergonomic issues, difficulty of access, provisional installation of other equipment, handling of loads or irreversible damage, as previously mentioned. Additionally, the motions of vessels may interfere with crew activities and well-being (Haward et al. 2009), which can change the operation of the measuring instrument itself, as well as the interpretation of the reading by the workers.

#### The function ‘monitor and interact with CCR control screens’

**Fig. 7:**
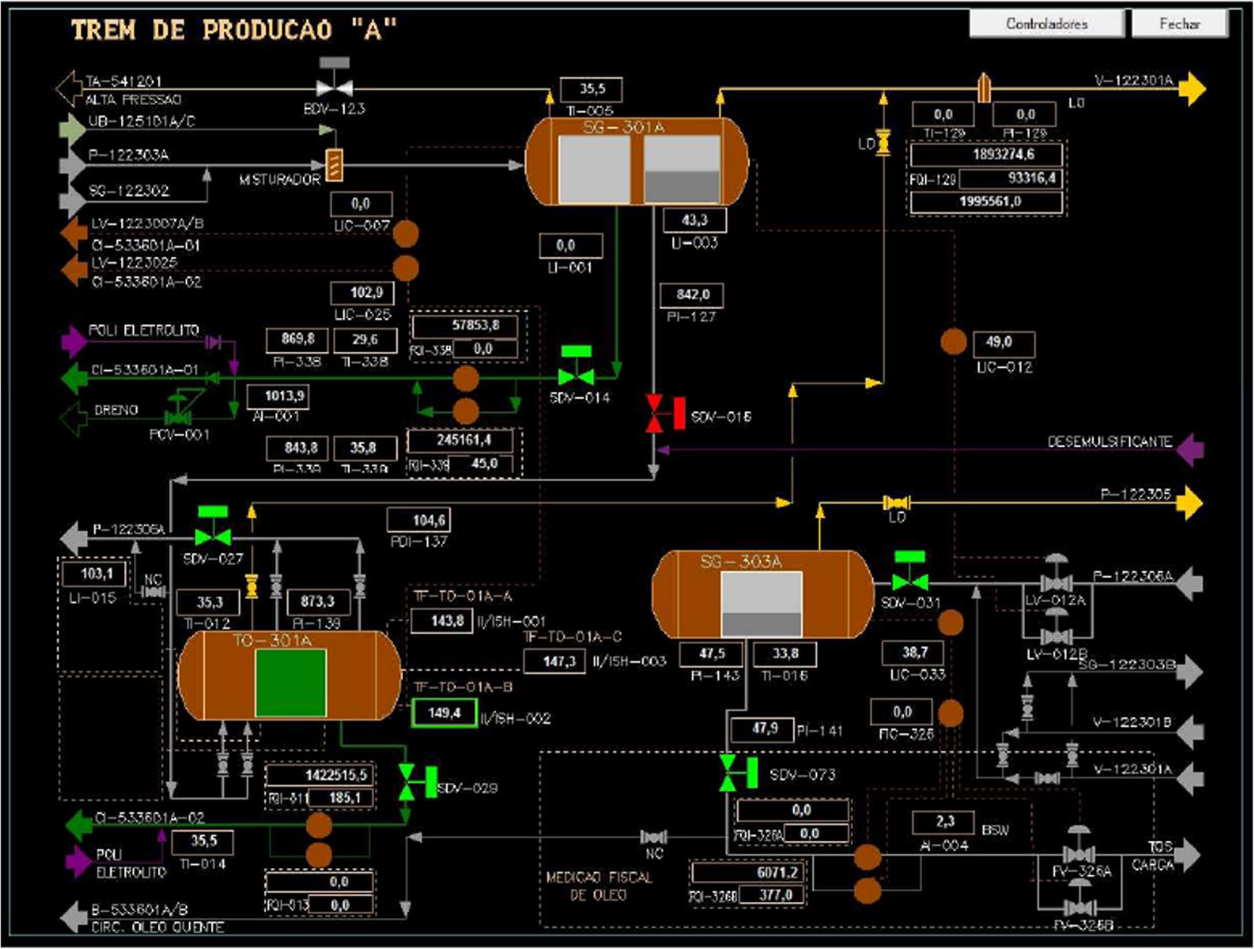
One of the several screens operated in CCR by FPSO operators of the oil treatment area. Source: J. França (2014)

The function ‘monitor and interact with CCR control screens’ presented the variabilities ‘too late’ for time and ‘imprecise’ for precision. Once this activity is performed inside the CCR, interacting with several screens at the same time, both the onboard observations and the interviews revealed that the operators experience a high cognitive (over)load. From the perspective of FRAM instantiations, the conditions of both cognitive underload and overload could be the source of variability in the output of the functions that are carried out by the human operator (Ferreira and Cañas 2019). Figure 7 presents only one of the many screens operated in the CCR by FPSO operators of the oil treatment area.

In offshore production platforms such as an FPSO, the control room is the core of its safe and efficient operation (Walker et al. 2014). The operation of this screen alone requires a considerable cognitive load, requiring specific competences from the operators. It is a complex task that relies on an ability to accurately monitor the entire system functioning as it unfolds over time; it requires being aware of alarms and signs that characterise a need for intervention (Stainer et al. 2021). This awareness is a competence that characterises situational awareness. In this specific case, it also interacts with decision-making, allowing operators to act on alarms and demands that are most critical, both in terms of time and response. Decision-making here is therefore subsidised by situational awareness and other competences of the operators; their mouse click on the CCR will act directly on the containment losses of the plant’s equipment. Consequently, such action on CCR screens is directly linked to the process safety of the entire FPSO since the decisions and interactions that take place here directly prevent primary losses of containment and catastrophic failures. At the same time, other functions also contribute to the FPSO’s process safety, such as ‘control containment losses in the process plant’ and the communication’s functions, which are represented by two different functions that complement each other.

#### The functions ‘proper communication between external area and CCR’, ‘proper communication between operations and other areas’ and ‘proper communication between offshore and onshore crew’.

**Fig. 8:**
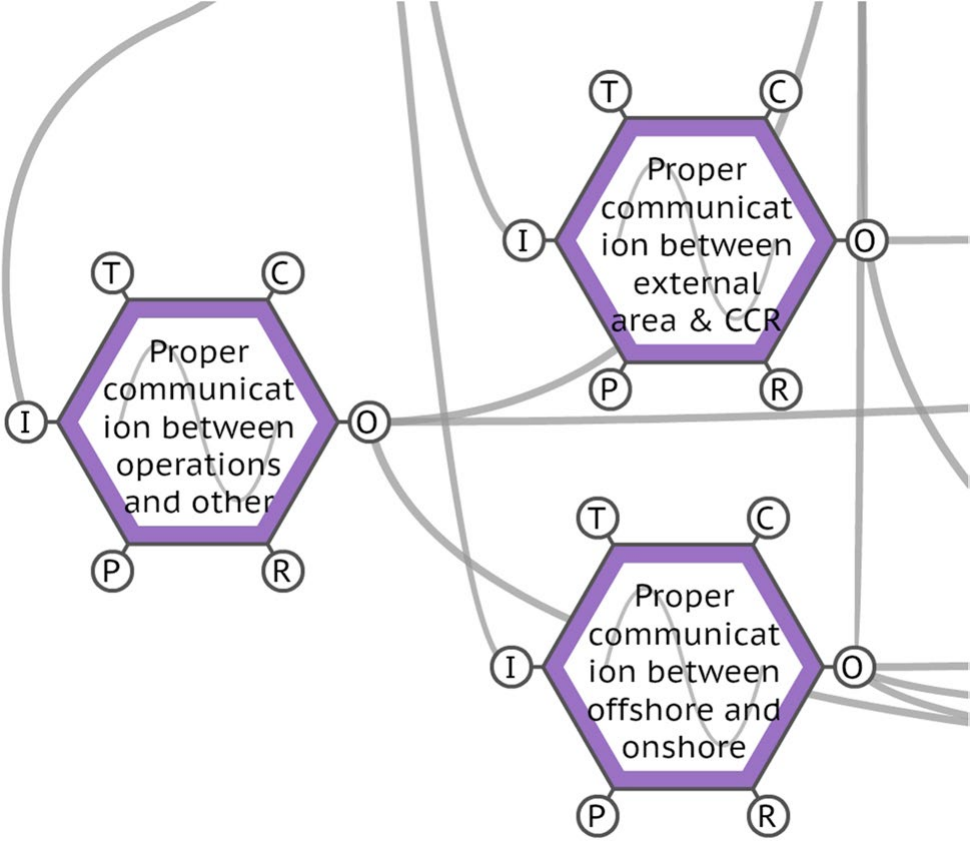
The FRAM functions regarding the communication process. Source: Authors (2020)

Although the functions ‘proper communication between external area and CCR’, ‘proper communication between operations and other areas’ and ‘proper communication between offshore and onshore crew’ presented variability only in precision: ‘acceptable’, these three functions play an important role, as being the resource aspect of three other key functions: ‘operating the oil treatment area of FPSO’, ‘control containment losses in the process plant’ and ‘have preparation for emergency situations in operations’. These functions are represented in Fig. 8.

The communication process in complex workplaces at sea, such as transportation ships and offshore oil platforms, is crucial for navigation, stability, onboard operations and the reliability of technological systems itself (B. Li and Su 2021). Communication is also a competence needed by operators, as it is the social mechanism responsible for binding all elements of the complex sociotechnical system that distinguishes the operational area. Humans are an inherently social species, with complex verbal and non-verbal communication abilities (Lieberman 2013); effective communication and teamwork, other non-technical skills, are essential for safe operations, especially in workplaces like an FPSO, where there are high-risk operations happening 24 h a day (Thomas 2018). Also, it is important to mention that communication is a competence that supports all the other non-technical skills, working metaphorically like cement in construction, providing the alloy and the necessary strength for the proper and safe performance of everyone, starting from the people and being reflected in the system.

As observed onboard and verified by operator interviews, verbal and non-verbal communication happens in a dynamic way – It is clear and assertive most of the time, but it can also be fuzzy and inaccurate. When the latter occurs in emergency and contingency situations, as represented by the function ‘have preparation for emergency situations in operations’, it can cause the opposite of preparation, contributing to a reaction chain of an accident, as observed with Deepwater Horizon and FPSO CSM. Particularly, the function ‘proper communication between offshore and onshore crew’ is the dynamic and active communication link between offshore operations and onshore support and management teams, in particular with maintenance and inspection teams. This function is the only one from the communication set that has coupling with the resource of ‘interaction with inspection and maintenance teams’, another key function of this system. The partnership between these teams is a critical element for the operation of the entire process plant, as it guarantees not only the regular working of the equipment but also the technological resources to respond to emergencies and guarantee a safety shutdown (Hart 2019). Indeed, the complexity, time pressure and harshness of offshore workplaces demand assertive and objective ways of communication, which is a key element for process safety and workers’ health (França and Hollnagel 2020). Since communication provides knowledge, institutes relationships, establishes predictable behaviour patterns, sustains attention and promotes integration, it is also a competence that leaves operators ready for expected and unexpected situations, contributing to the resilience of the whole system.

#### The function ‘perform daily checklist inspection of external area’

**Fig. 9:**
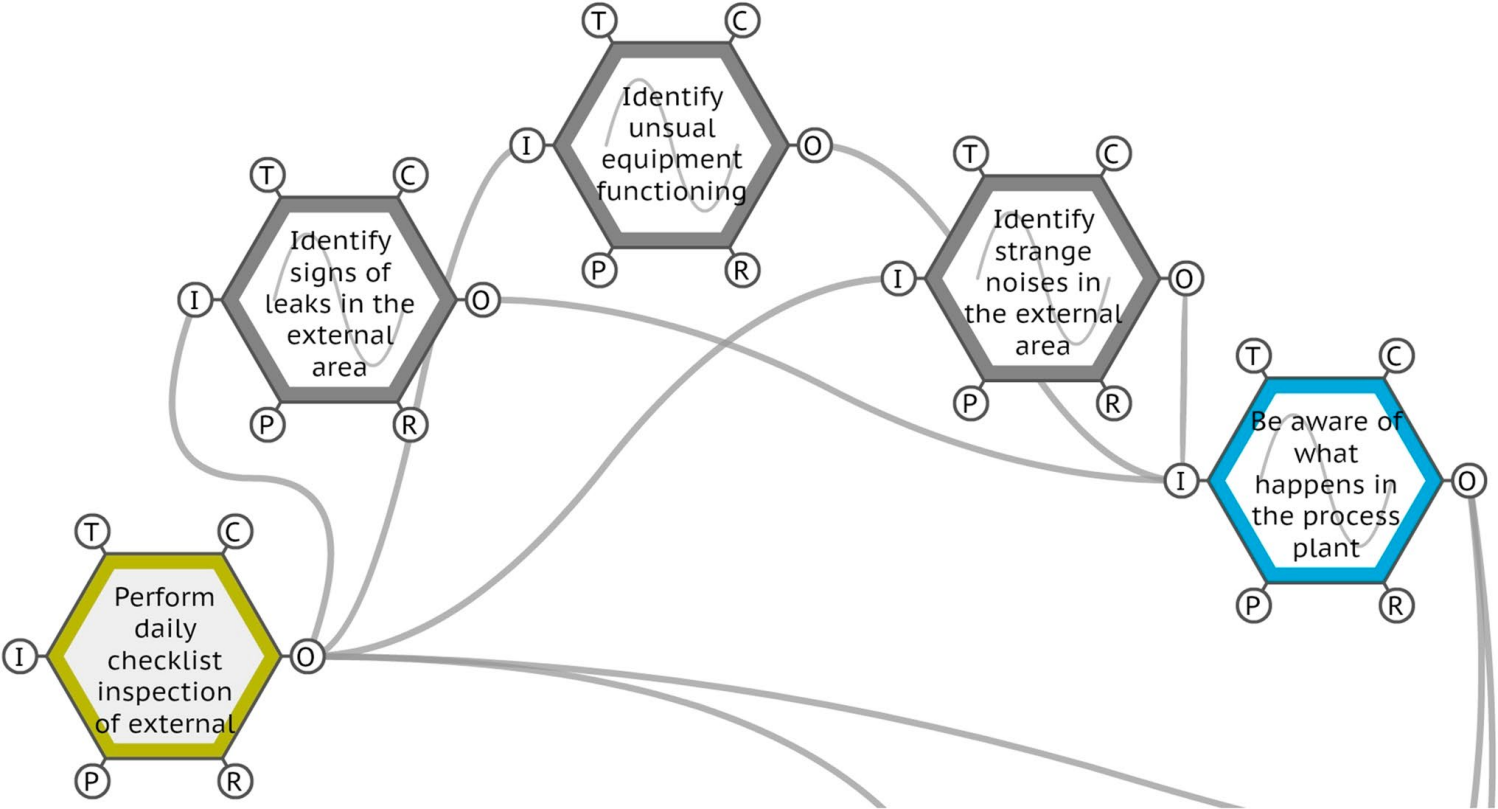
Extract from the main model for the function ‘perform daily checklist inspection of external area’. Source: Authors (2020)

The function ‘perform daily checklist inspection of external area’ presented the variabilities ‘too late’ for time and ‘acceptable’ for precision. The onboard observations and interviews demonstrated that the daily checklist presents incoming constraints that cannot be met most of the time due to multitasking demands. Compared with the other activities in the external area, the daily checklist could be considered the lightest in terms of physical effort because it mainly consists of walking around the external area, inspecting processes, equipment and the environment, guided by a checklist. However, in terms of cognitive and social effort, it is intense and varied, as it is necessary to recognise any signs, sounds or behaviours that signal an unusual functioning of the whole system. One of the operators declared that it is during his routine checklist when he ‘feels’ the plant and ‘talks’ to the equipment, interacting so intimately and intensely that he can perceive even minimal changes in the field. This perception, as can be seen in the extract from the main model using FMV® (Hill 2018) in Fig. 9, allows for an awareness of what is happening in the process plant.

The function ‘perform daily checklist inspection of external area’ outputs are ‘identify signs of leaks in the external area’, ‘identify strange noises in the external area’ and ‘identify unusual equipment functioning’; these are also the inputs for ‘be aware of what happens in the process plant’, which is a key competence for operating the process plant. This daily inspection of the external area is thus a crucial activity for process safety, as it not only provides and maintains an awareness of the process plant’s functioning but also enables the possibility of recognising and acting in a loss of primary containment (LOPC), preventively intervening in the losses of contention that could potentially cause process accidents. In this respect, the human element of every system – the workers – is in fact an effective (and dynamic) safety barrier, drawing on their competences, such as situation awareness, communication and decision-making, and provides the ability to adjust and protect themselves and the entire process plant.

Indeed, the onboard observations revealed that worker competences are part of the real work done by the operators, helping them adjust their performance to respond to the system’s demands. This ability of a sociotechnical system to adapt and create a successful outcome in everyday operations highlights the well-known difference between the WAD and the WAI, showing that the recognition and comprehension of these worker competences create a path that can simultaneously increase performance and safety since both are based in the variabilities present in the everyday activities performed by humans. Especially in the function ‘perform daily checklist inspection of external area’, it is possible to see that a real action in the field – the checklist – provides feedback in a chain of interactions that recognises the finite elements of the process – leaks, noises and functioning – and intrinsically develops a human competence – situational awareness – that is part of human performance and increases system safety.

#### The function ‘interaction with inspection and maintenance teams’

**Fig. 10:**
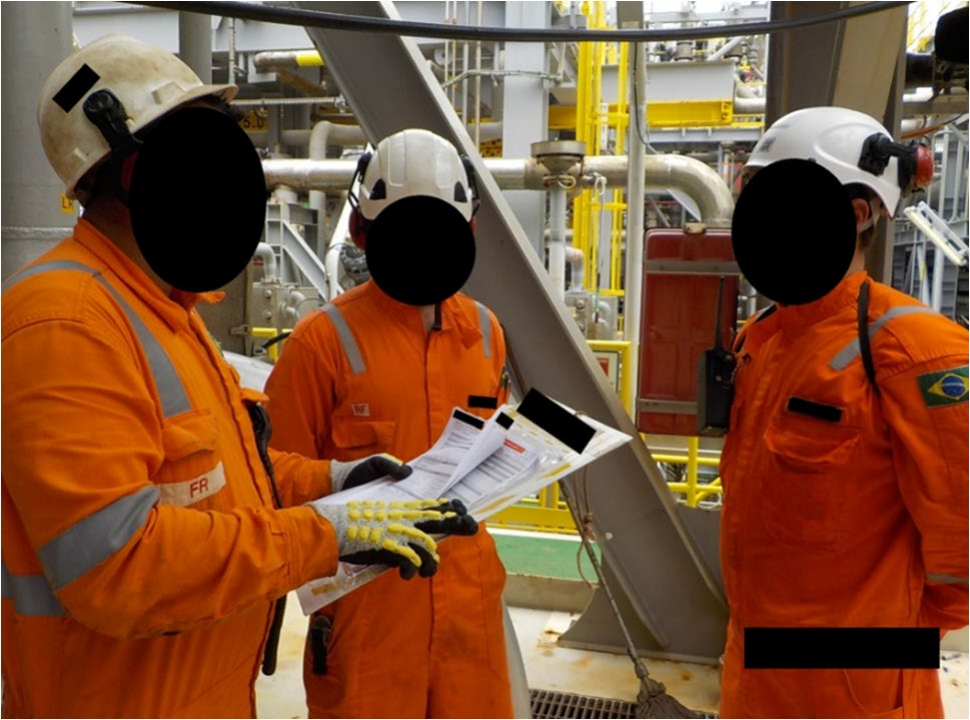
Interaction with a maintenance team in the oil treatment external area. Source: Authors (2020)

The function ‘interaction with inspection and maintenance teams’ presented the variabilities ‘too late’ for time and ‘acceptable’ for precision. From the first moment of the work, with the starting function ‘have a new workshift of production operators’, to the last moment of the shift, the operators actively interact with other teams – notably the teams from the maintenance, instrumentation and inspection department. The work on ships, in offshore platforms and on offshore wind farms, is characterised by narrow workplaces and a high degree of interaction with different teams from different areas together, keeping the system running. The work activities in such facilities in the sea depend on mutual adjustments of their parts and interactions to make optimal use of the available resources while maintaining a minimum safety separation (van Westrenen and Praetorius 2014). Therefore, these adjustments fall on the workers, who have to develop specific competences that enable such performance. The onboard observations and the FRAM model show that communication, teamwork and situational awareness are real examples of these competences, enabling a dynamic behaviour of preparedness that is present in the individual interactions with the system as much as when interacting with other workers and groups from maintenance, instruments and inspection areas. Figure 10 presents an example of the interaction with a maintenance team in a moment of work permit (WP) emission and authorization in the oil treatment external area of the FPSO.

Particularly in the O&G offshore area, investigations and analysis of several accidents have pointed to a lack of communication between crew members, showing the importance of specific competences onboard offshore platforms (Abimbola et al. 2014). In drilling platforms, for instance, the need for proper and objective communication between the driller and the drill floor – including non-verbal communication through eye contact – is essential to perform a job safely (França et al. 2020). These evidence from literature, observations and the FRAM model show that communication, teamwork and situational awareness are present competences in the interaction between the FPSO operators and other teams, contributing to productive and safe work in maintenance, instrumentation and inspection.

### Human competences in onboard activities of offshore facilities

Considering the findings of this research and the scientific background, there are specific human competences observed in the onboard activities of workplaces at sea that are part of worker performance in dealing with all system demands, requiring simultaneous preparedness for daily activities and emergency situations. These have been studied in other domains, including civil aviation (Crichton 2017), electrical power grid (Wachs et al. 2012) and air traffic control (Carvalho 2011), involving debate about the appropriate recognition, implementation and training. Despite this debate, all studies converge in recognising these skills as being essential for the development of a safe and productive workplace. Specific individual competences in the healthcare area had been found as essential for routine and emergency demands (Arbelaez-Garces et al. 2018). In the O&G industry, these competences are well known and classified as non-technical skills (Flin et al. 2016), with recognition, analysis and development as a strategic part of safety and health actions onboard. These non-technical skills enable workers to be in a dynamic state of preparation, meeting the demands of the complex sociotechnical systems that form the workplaces of the O&G facilities at sea (IOGP 2020). External pressure from geopolitics, changes in energy demands and rush deadlines from contractors adds greater complexity to this scenario, requesting even more from the set of technical and non-technical skills of the offshore workers. To be ready for this, proper preparation is needed.

The testimony of several operators states that to perform daily operations and be ready for emergency situations, it is crucial to have proper training. In terms of daily operations, this training enables the operators to be aware of when something is behaving abnormally or providing different signals (noises, smells, malfunctioning, etc.), to identify what is happening before any loss occurs and to simultaneously act to prevent LOPC that could start a major accident. In other words, the combination and dynamic integration of individual characteristics and the technical and non-technical skills promoted by training are responsible for the operator’s performance and behaviour in managing their daily work activities and preparing for emergencies. Thus, their work is simultaneously responsible for the productivity and safety of the operations at the process plant, effectively contributing to process safety.

This study enables an expanded comprehension of the complexity in the onboard work activities observed, as the FRAM allows a systematic and thorough analysis, considering connections between functions in terms of time, preconditions, resources and control, in addition to the traditional input and output, enabling the perception of various dimensions of system interactions. Behaviour, attitudes, technical and non-technical skills, integrated with all other elements of the system, and embedded in the company’s organisational culture, form a complex sociotechnical system in the oil treatment of the FPSO, being then evidenced and analysed by the FRAM model developed. With that, it was possible to perceive how the actions of operators in the sharp end of the production plant influence the entire process, thus being responsible for safety, productivity and preparation in emergency situations.

## Conclusions

This study found a relationship between specific human competences, safety and complexity in the interactions of the everyday work in the oil treatment operations of an FPSO. The analysis was enabled by an FRAM model that demonstrated how individual variabilities resonate in the system, highlighting the foreground and background functions that are essential for an operator’s performance in their daily work, as well as the preparedness during contingency circumstances. This model was built and validated by the operators, offshore experts and FRAM specialists. Besides the well-known non-technical skills presented in the O&G literature, such as teamwork and communication, the operators have developed specific skills to deal with the unique and complex demands of their offshore work, using the natural variability of their behaviour in a positive way. The identification of leaks and strange noises in the external area and signs of unusual equipment functioning enable situational awareness of what is happening in the operational area, but it is the workers’ individual competences that are necessary for daily performance. These individual competences – technical and non-technical skills – were observed in the communication process, and through the FRAM model, has been coupling activities to enable several operations onboard, such as the monitoring of CCR control screens and the daily checklist inspection of external area. This analysis highlights the operator´s ability to monitor, respond to, adapt, and cope with the system’s demands, giving appropriate and safe responses and making everyday operations possible and productive. Indeed, it is possible to see that, in this context, the worker is actually the solution – not the problem – for all the complex demands of workplace interactions.

## Data Availability

All data of this research are directly available within this publication.
